# Essential Roles of the Histone Demethylase KDM4C in Renal Development and Acute Kidney Injury

**DOI:** 10.3390/ijms23169318

**Published:** 2022-08-18

**Authors:** Heng-Chih Pan, Yau-Hung Chen, Wei-Ching Fang, Vin-Cent Wu, Chiao-Yin Sun

**Affiliations:** 1 Graduate Institute of Clinical Medicine, College of Medicine, National Taiwan University, Taipei 10607, Taiwan; 2 College of Medicine, Chang Gung University, Taoyuan 33302, Taiwan; 3 Division of Nephrology, Department of Internal Medicine, Keelung Chang Gung Memorial Hospital, Keelung 20401, Taiwan; 4 Community Medicine Research Center, Keelung Chang Gung Memorial Hospital, Keelung 20401, Taiwan; 5 Department of Chemistry, Tamkang University, Tamsui, New Taipei City 25137, Taiwan; 6 Department of Family Medicine, Linkou Chang Gung Memorial Hospital, Taoyuan 333423, Taiwan; 7 Division of Nephrology, Department of Internal Medicine, National Taiwan University Hospital, Taipei 100229, Taiwan; 8 NSARF (National Taiwan University Hospital Study Group of ARF), TAIPAI, (Taiwan Primary Aldosteronism Investigators), and CAKS (Taiwan Consortium for Acute Kidney Injury and Renal Diseases), Taipei 100229, Taiwan

**Keywords:** acute kidney injury, autophagy, ischemia reperfusion kidney injury, KDM4C, mitochondria

## Abstract

Background: Lysine demethylase 4C (KDM4C) is a nuclear protein that is essential for histone modification and acts as an important regulator of several transcription factors. Previous studies have shown that KDM4C may also play a role in mediating stress responses. The purpose of this study was to examine the roles of KDM4C in kidney development and acute kidney injury (AKI). Methods: The effect of KDM4C on kidney development was assessed by comparing the kidney phenotype between 96 zebrafish embryos treated with kdm4c-morpholino oligonucleotide and 96 untreated zebrafish embryos. We further examined whether KDM4C is essential for maintaining cell survival in AKI. Cultured human renal tubular cells were used for the in vitro study. Wild-type and Kdm4c knockout mice (C57BL/6NTac-Kdm4ctm1a(KOMP)Wtsi) were divided into a sham group and model group, and then subjected to ischemic reperfusion kidney injury (IRI-AKI). Blood samples and kidneys were collected at different time points (day 3, day 7, day 14, and day 28) and were processed for in vivo studies (*n* = 8 in each group). Results: Kdm4c knockdown significantly decreased zebrafish embryo survival and impaired kidney development. The in vitro study showed that KDM4C inhibition by JIB04 significantly increased cellular apoptosis under oxidative stress conditions. KDM4C knockdown cells had impaired autophagy function under stress conditions. The IRI-AKI mice study showed that KDM4C protein levels dynamically changed and were significantly correlated with HIF-1α levels in AKI. Kdm4c^−/−^ mice had significantly more severe renal impairment and increased kidney fibrosis than the wild-type mice. Cytokine array results also indicated that the kidneys of Kdm4c^−/−^ mice had increased inflammation in AKI compared with the wild-type mice. Further RNA sequence analysis revealed that KDM4C may regulate transcription factors related to mitochondrial dynamics and function. Conclusions: Our study suggests that KDM4C may play a critical role in regulating mitochondria, which is related to a protective effect on maintaining cell survival in AKI.

## 1. Introduction

Lysine demethylase 4C (KDM4C) is a nuclear protein that can modify histone by specifically demethylating Lys-9 and Lys-36 residues of histone H3 [1]. Investigators have demonstrated that KDM4C is critical for efficient cancer cell growth and that it is expressed in colorectal, lung, breast, prostate, and other tumor cells [2]. KDM4C acts as an important regulator of several transcription factors, including androgen and estrogen receptors [3]. In addition, KDM4C has been reported to, not only modify histones, but also regulate gene expression by directly interacting with transcription factors and changing their activities [4].

KDM4C has been shown to interact with activating transcription factor 4 (ATF4), to target serine pathway genes and activate transcription. In addition, under hypoxic stress conditions, the expression of KDM4C has been shown to be upregulated by gene transcription activation of hypoxia inducible factor-1 (HIF-1) [5]. Given that KDM4C has been shown to regulate the activities of transcription factors, including HIF-1 and ATF42, 4, and 5, KDM4C may play a role in mediating stress responses.

Ischemic reperfusion injury (IRI) is the leading cause of acute kidney injury (AKI) [6]. It results in acute tubular necrosis and apoptosis, accompanied by the rapid infiltration of polymorphonuclear leukocytes, lymphocytes, and macrophages into the interstitium. These cells are believed to be attracted to the injured kidney in response to inflammatory cytokines produced locally by cells, such as resident dendritic cell and injured tubular and endothelial cells [7]. Persistent inflammation in the kidneys is thought to contribute to the development of tubulointerstitial fibrosis with functional impairment.

Autophagy is a strictly regulated lysosomal degradation pathway under stress conditions, such as hypoxia, oxidative stress, endoplasmic reticulum stress, and deoxyribonucleic acid (DNA) damage [8,9]. It removes unnecessary or dysfunctional cell components and allows for the recycling of cellular components [10]. Autophagosome formation is tightly regulated by a series of autophagy-promoting proteins [11]. Beclin-1 and LC3 are responsible for initiation of autophagosomes, as well as the extension and closure of autophagosome double membranes, respectively [12].

The number of autophagosomes and autolysosomes can be reflected by lipidated LC3-II, which is formed by the conjugation of LC3 and phosphatidylethanolamine [13]. Autophagy is not only regulated at the post-translational level, DNA methylation and histone modifications have also been shown to be highly involved in autophagy regulation [14]. Previous studies have indicated that autophagy is crucial for the cellular homeostasis of renal tubules and is cytoprotective in response to AKI9.

Although the role of KDM4C in AKI is largely unknown, it has been reported to be involved in cell signaling, the cell cycle, and cell growth [15]. The bifunctional roles of KDM4C in histone modification and direct interactions with transcription factors also support that KDM4C plays crucial roles in regulating kidney injury [16]. However, the precise role of KDM4C in AKI has yet to be elucidated.

Numerous developmental-related genes play roles in kidney repair after AKI [17]. In the current study, we first used morpholino oligonucleo-tide (MO) to inhibit kdm4c and examine the changes in the kidney development of zebrafish. Then, a IRI-AKI mice model was further used to investigate the roles of KDM4C in the pathogenesis of AKI.

## 2. Results

### 2.1. Zebrafish Study

#### The kdm4c Gene Is Essential for Zebrafish Embryo Survival and Kidney Development

**Figure 1:**
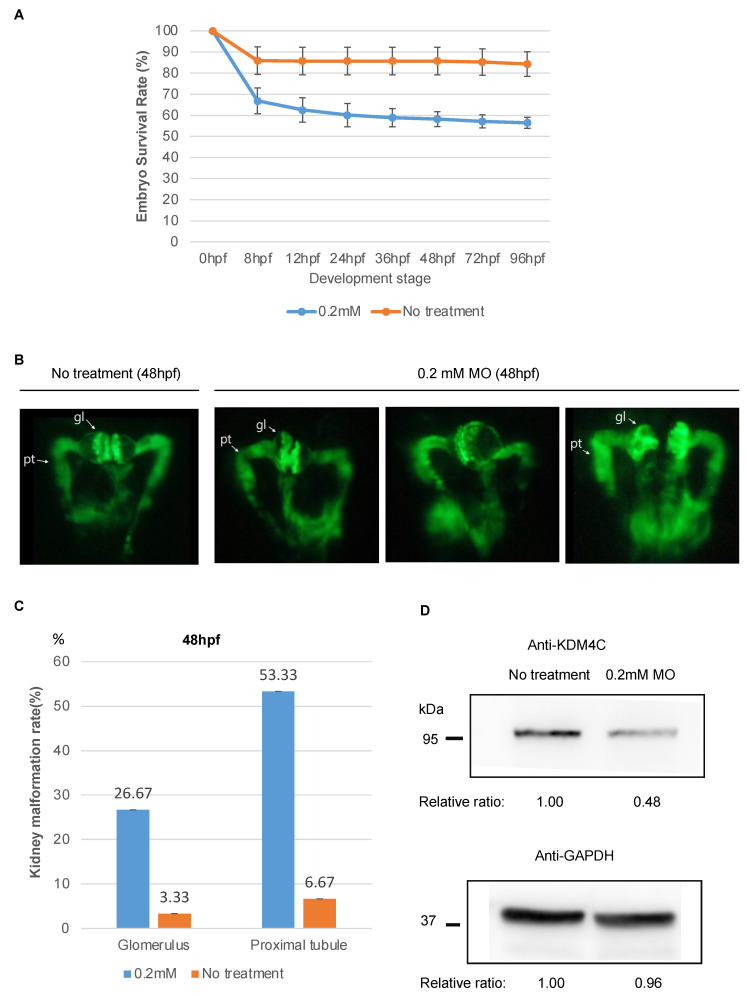
Embryo survival and kidney phenotypes of zebrafish embryos (96 untreated embryos and 96 embryos treated with kdm4c-MO injection). (**A**). Comparison of embryo survival rate between embryos treated with a single injection of 0.2 mM kdm4c-MO and untreated embryos (n = 96 in each group). (**B**). Pronephric phenotypes of zebrafish embryos after kdm4c-MO injection. Each kidney photo was taken from the dorsal view and at the developmental stage of 48 hpf of bTg(wt1b:EGFP) embryos. Compared with the untreated group, after kdm4c-MO injection, the renal tubules were tortuously dilated and the glomeruli were swollen. (**C**). Comparison of the defect rates of morphological phenotypes (tortuously dilated renal tubules and swollen glomeruli) of zebrafish embryos between untreated group and kdm4c-MO injection group. (**D**). Results of Western blotting for Kdm4c with zebrafish embryo kidneys with or without kdm4c-MO injection. Abbreviation: KDM4C, Lysine demethylase 4C; MO, morpholino oligonucleotides.

To assess the function of *kdm4c* during zebrafish kidney development, we knocked down the expression of *kdm4c* using *kdm4c*-MO, designed to bind to the region encompassing the start codon of vhl and block its translation. The results showed that *kdm4c* knockdown significantly decreased zebrafish embryo survival from the development stage of 8 hpf to 96 hpf (*n* = 96 in treated group and untreated group, respectively) (Figure 1A).

At 48 hpf, embryos derived after injecting *kdm4c*-MO had a more malformed kidney phenotype than the non-injected control group (Figure 1B,C). Distended pronephric tubules and swollen glomeruli are shown. Western blotting for Kdm4c in the kidneys of zebrafish embryos with or without *kdm4c*-MO injection are shown in Figure 1D.

### 2.2. Human Cell Study

#### 2.2.1. KDM4C Inhibition Decreased Cellular Apoptosis under Oxidative Stress In Vitro

**Figure 2:**
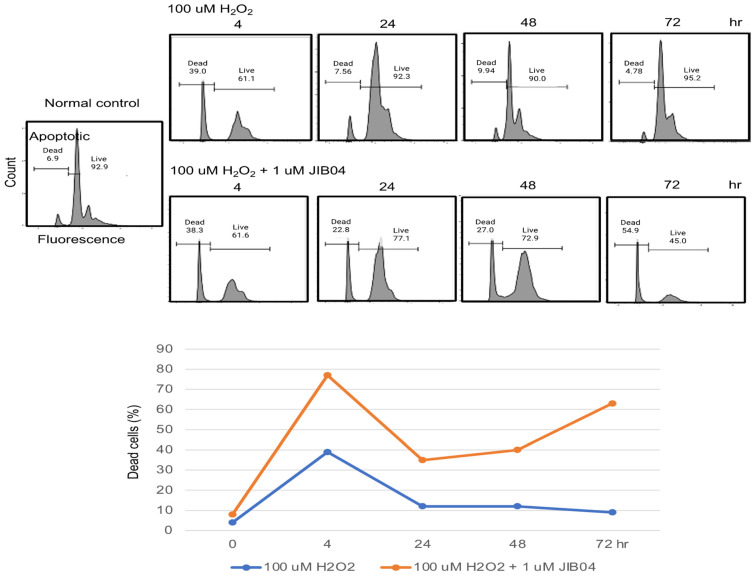
KDM4C inhibition increased cell apoptosis during oxidative stress in vitro. HEK293 cells treated with the KDM4C inhibitor, JIB04 (1 μM) were used for the study. H_2_O_2_ (100 μM) treatment was used to induce oxidative stress. Cell apoptosis was analyzed by flow cytometry at the time points indicated in the figure. The percentages of dead cells at different time points after treatment are shown. Abbreviation: KDM4C, Lysine demethylase 4C.

JIB04 (NSC 693627) is a selective Jumonji histone demethylase inhibitor [18]. The results demonstrated that KDM4C inhibition by JIB04 significantly increased cellular apoptosis under oxidative stress conditions (Figure 2). The percentages of dead cells significantly increased in cultured HEK293 cells treated with JIB04 during the course of oxidative stress.

#### 2.2.2. KDM4C Depletion Impaired Autophagy In Vitro

Autophagy is a key protective mechanism for cell survival under stress conditions [19]. In this study, we used serum starvation conditions to activate autophagy in vitro [20]. The results showed that serum starvation significantly increased autophagy marker protein expressions, including cleaved LC3B, BCL1, and BCL2. In HEK293 cells with KDM4C depletion, cleaved LC3B was not observed after serum starvation.

**Figure 3:**
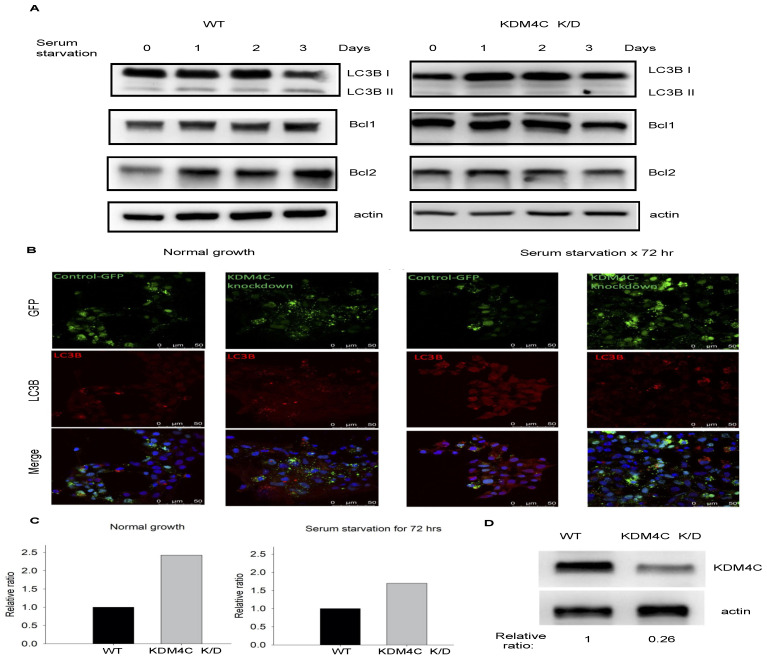
KDM4C depletion impaired autophagy during serum starvation in vitro. HEK293 cells transfected with *KDM4C*-sh plasmid were used for the study. Cells were harvested after serum starvation at the time points indicated in the figure. (**A**). Results of Western blotting for autophagy markers, LC3B, BCL1, and BCL2. (**B**). Results of immunofluorescence staining for LC3B in control and *KDM4C* knockdown HEK293 cells with or without serum starvation. (**C**). Relative ratio of fluorescence intensities of GFP-LC3B puncta with WT as the denominator. (**D**). Results of Western blotting for KDM4C in control and *KDM4C* knockdown cells. Abbreviation: K/D, knock down; KDM4C, Lysine demethylase 4C; WT, wild-type.

In addition, BCL1 and BCL2 significantly decreased after 3 days of serum starvation (Figure 3A).

The immunofluorescent staining results also showed that KDM4C depletion significantly decreased LC3B puncta formation after serum starvation (Figure 3B,C). The relative levels of KDM4C in wild-type and *KDM4C* knockdown cells are demonstrated in Figure 3D. These findings suggest that *KDM4C* knockdown may impair autophagy under stress conditions.

### 2.3. Mice Study

#### 2.3.1. Dynamic Changes of KDM4C Protein Levels in AKI

**Figure 4:**
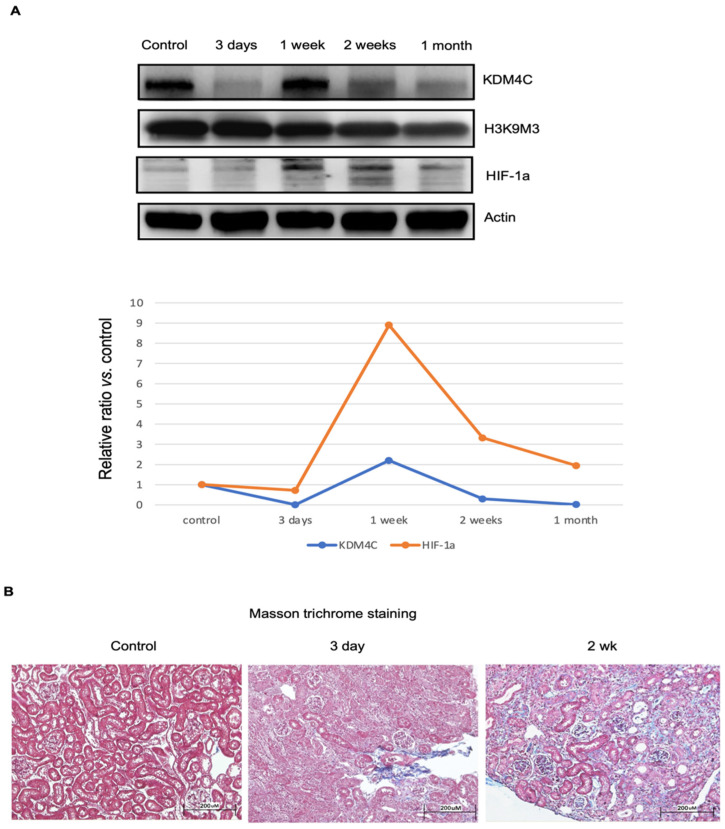
Temporal changes in KDM4C and progression of kidney fibrosis during kidney injury. Wild-type mice receiving kidney ischemia-reperfusion injury (IRI) were used for this study. Kidney tissues, after IRI for the durations indicated in the figure, were harvested (*n* = 8 for each group). (**A**). Western blotting results for KDM4C, H3K9M3, and HIF-1α. The average relative ratios of KDM4C and HIF-1α along the course of injury are shown. (**B**). Masson trichrome staining results of kidney tissues after IRI. Mild glomerulous and tubulointerstitial fibrosis, 3 days after receiving IRI-AKI. The severity of glomerulous and tubulointerstitial fibrosis was even more pronounced 2 weeks after IRI-AKI. Abbreviation: HIF, hypoxia-inducible factor; KDM4C, Lysine demethylase 4C.

Mice with kidney IRI were used for this study (*n* = 8 in each group). The results showed that KDM4C protein levels dynamically changed in AKI, and that AKI significantly decreased kidney KDM4C expression (3 days after IRI). At 1 week after IRI, the KDM4C levels recovered, but they then decreased again, 2 to 4 weeks after IRI. HIF-1α is a master transcription factor responsible for kidney repair in AKI [21]. The Western blot results demonstrated that the dynamic changes in KDM4C levels significantly correlated with HIF-1α levels (Figure 4A). However, no significant changes in kidney H3K9M3 protein were noted during the course of injury (Figure 4A). Representative Masson trichrome staining results of kidney tissues after ischemia-reperfusion injury are shown in Figure 4B.

#### 2.3.2. KDM4C Deletion Increased the Severity of AKI

**Figure 5:**
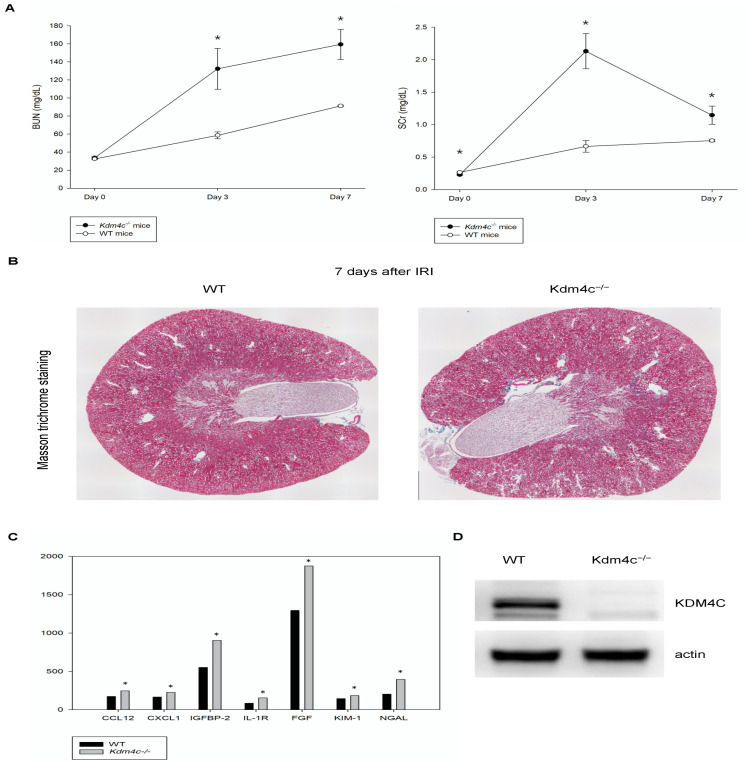
KDM4C depletion worsened acute kidney injury. Study animals receiving kidney ischemia-reperfusion injury were used for this study. Kidney tissues and serum after ischemia-reperfusion injury for the durations indicated in the figure were harvested (*n* = 8 for each group). (**A**). Serum BUN and creatinine levels of wild-type and Kdm4c^−/−^ mice after kidney ischemia-reperfusion injury. (**B**). Masson trichrome staining results of kidney tissues of wild-type and Kdm4c^−/−^ mice after ischemia-reperfusion injury. (**C**). Representative results of kidney inflammatory cytokines of the study animals, 1 week after ischemia-reperfusion injury. (**D**). Results of Western blotting for KDM4C in the kidneys of wild-type and Kdm4c^−/−^ mice at the age of 10 weeks. Abbreviation: BUN, blood urea nitrogen; CCL12, C-C motif ligand 12; CXCL1, C-X-C motif ligand 1; FGF, fibroblast growth factor; IGFBP-2, insulin-like growth factor binding protein-2; IL-1R, interleukin-1 receptor; KDM4C, lysine demethylase 4C. KIM-1, kidney injury molecule-1; NGAL, neutrophil gelatinase-associated lipocalin; WT, wide-type. * *p* < 0.05.

Wild-type and *Kdm4c^−/−^* mice with ischemia-reperfusion kidney injury were used for this study (*n* = 8 in each group). Serum BUN and creatinine analysis revealed that the *Kdm4c^−/−^* mice had higher serum BUN and creatinine levels than the wild-type mice after acute kidney injury (Figure 5A). Representative Masson trichrome staining results of kidney tissues after ischemia-reperfusion injury are shown in Figure 5B, which demonstrated that the kidneys of the *Kdm4c^−/−^* mice had increased fibrosis compared with the kidneys of the wild-type mice.

Neutrophil gelatinase-associated lipocalin (NGAL) and kidney injury molecule-1 (KIM-1) are markers of renal tubular injury [22,23], and insulin-like growth factor binding protein-2 (IGFBP-2) is also a novel marker for kidney inflammation [24]. The cytokine array results of kidney protein lysates showed that the levels of NGAL and IGFBP-2 in the kidneys of *Kdm4c^−/−^* mice were 1.5-fold higher than those of WT mice, which indicated that *Kdm4c^−/−^* mice had increased kidney inflammation after AKI compared with wild-type mice. Representative results of kidney inflammatory cytokines of the study animals were shown in the Figure 5C and Supplementary Figure S2. The relative levels of KDM4C in the kidneys of the wild-type and *Kdm4c^−/−^* mice are demonstrated in Figure 5D. These results suggested that KDM4C may play a critical role in AKI.

#### 2.3.3. Differential Gene Expressions in the Kidneys of the Wild-Type and Kdm4c^−/−^ Mice

**Figure 6:**
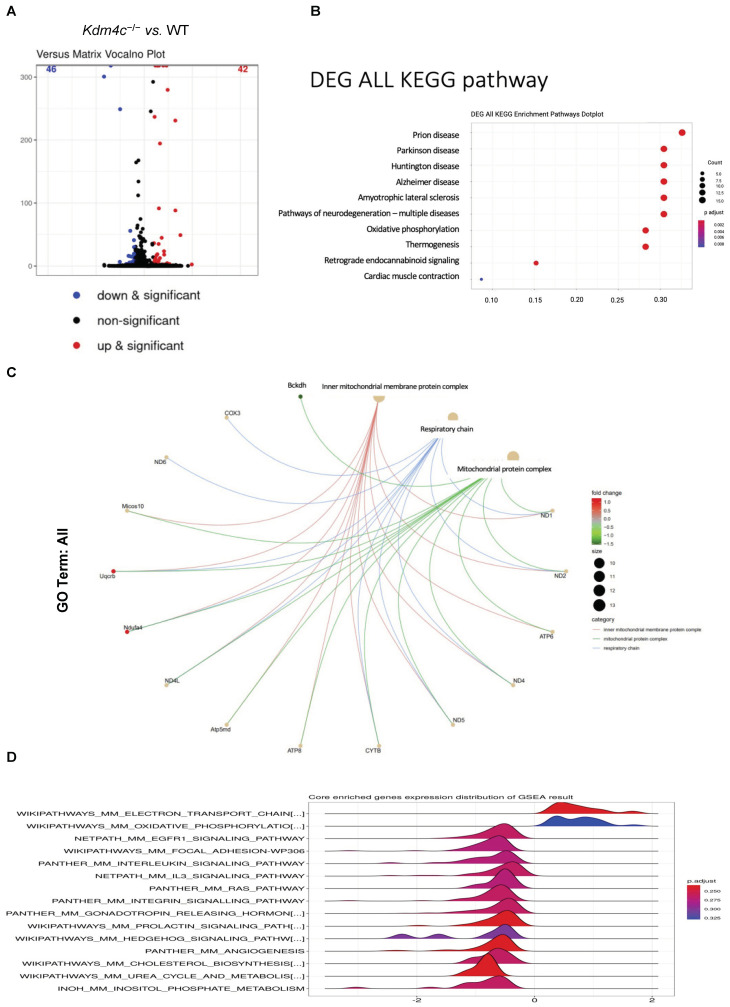
Differential kidney expressions of genes between the wild-type and Kdm4c^−/−^ mice. Kidney tissues of wild-type and Kdm4c^−/−^ mice at the age of 10 weeks were used for this study (*n* = 8 in each group). (**A**). Volcano plot showing differential kidney gene expressions between the wild-type and Kdm4c^−/−^ mice. The results showed that 42 genes had higher expression levels, while 46 genes had lower expression levels in the Kdm4c^−/−^ mice than in the wild-type mice (*p*-value < 0.01, *q*-value < 0.01). (**B**). The KEGG pathway analysis results showed that Kdm4c deletion was also associated with a variety of neurodegenerative diseases and metabolic diseases. (**C**). GO enrichment analysis showed that the Kdm4c^−/−^ mice had significantly different expressions in several pathways, including inner mitochondrial membrane protein complex and mitochondrial protein complex. (**D**). GSEA revealed that Kdm4c deletion was also associated with several pathways involved in mitochondrial function, including mitochondrial electron transport chain, oxidative phosphorylation, and mitochondrial membrane phospholipid-based signal pathways. Abbreviation: KDM4C, lysine demethylase 4C; KEGG, Kyoto Encyclopedia of Genes and Genomes; GSEA, gene set enrichment analysis; WT, wild-type.

Our results suggested that KDM4C is critical for kidney development and autophagy activation under stress conditions. We further investigated whether deletion of KDM4C was correlated with impaired cellular metabolic homeostasis. An RNA expression study, used to compare the gene expression levels between 10-week old wild-type and *Kdm4c^−/−^* mice under normal growth conditions (*n* = 8 in each group), showed that 42 genes had higher expression levels, while 46 genes had lower expression levels in the *Kdm4c^−/−^* mice than in the wild-type mice (*p* < 0.01) (Figure 6A). Further analysis revealed that KDM4C was involved in mitochondrial inner membrane protein complex, mitochondrial protein complex, mitochondrial electron transport chain, oxidative phosphorylation, and mitochondrial membrane phospholipid-based signal pathways, and correlated with a variety of neurodegenerative diseases and metabolic diseases (Figure 6B–D).

These findings suggested that KDM4C may be involved in mitochondrial dynamics and function. Previous studies have shown that HIF-1, LC3B II, LC3, BCL2, Beclin-1, and ATG5 are involved in the regulation of mitochondrial metabolism. Our results support that KDM4C may potentially interact with some transcription factors and play a role in the regulation of mitochondrial dynamics and function.

## 3. Discussion

In the present study, we demonstrated that KDM4C is essential for kidney development and plays a role in renoprotection in AKI. KDM4C deletion was related to the aggravation of AKI. Under stress conditions, *KDM4C* knockdown HEK293 cells had decreased expressions of LC3B, BCL1, and BCL2, suggesting that KDM4C may be involved in autophagy pathways. Moreover, we also found that KDM4C played a role in mitochondrial function.

Autophagy is essential for maintaining cell homeostasis [25]. Several protein complexes of autophagy-related proteins (Atg) are involved in the regulation of autophagy in different phases [26,27]. The Atg14L-Beclin 1-Vps34-Vps15 complex can regulate the initiation of autophagy and the maturation of autophagosomes [28]. In addition, the Atg5–Atg12/Atg16 complex and microtubule-associated protein 1 light chain 3–phosphatidyl ethanolamine (MAP1LC3/LC3–PE) can regulate the extension and completion of autophagosomes [29]. In the kidney, autophagy plays a very important role in podocyte differentiation, and it plays a crucial role in the Notch signaling pathway in the process of kidney development [11].

Our results showed that *KDM4C* knockdown HEK293 cells had lower expressions of LC3B I, LC3B II, BCL1, and BCL2 than wild-type cells under starvation. In addition, the inhibition of *KDM4C* was correlated with increased cellular apoptosis under oxidative stress conditions. These findings suggest that the deletion of KDM4C is associated with impaired autophagy in the kidneys, which may explain our finding that a lack of *KDM4C* gene resulted in failure of kidney development and embryo survival.

Previous studies have provided substantial evidence that autophagy is deeply involved in the regulation of inflammation and the biology of immune cells [30]. The inflammatory response is designed to control pathogen invasion and initiate tissue repair. The absence of intact autophagy function may lead to impaired cellular metabolism and inability to activate the immune system [30]. Basal autophagy is vital for the turnover of damaged organelles in renal proximal tubules in normal physiological conditions [31,32].

As demonstrated in this study, the expression levels of numerous inflammatory cytokines, as well as AKI biomarkers, were increased in the *Kdm4c^−/−^* mice, and renal damage was augmented in these mice when subjected to IRI. HIF-1α is an important transcription factor in the cell survival response to hypoxia, by regulating HIF target genes, including microRNAs [21]. Accumulating evidence has indicated that the protective effect of HIF-1α against hypoxic-ischemic injury might involve autophagy activation. Previous studies have demonstrated that HIF-1α regulates autophagy by interacting with stress response transcription factors, such as BCL1, BCL2, and mTOR [33,34,35]. Our results further indicated that the expression level of KDM4C was significantly correlated with that of HIF-1α in AKI, which suggests that KDM4C may also play a role in mediating the stress response in AKI.

Mitochondrial homeostasis is important for maintaining kidney function because of the high oxygen consumption of the kidneys [36]. Mitochondrial pathology disrupts oxidative phosphorylation and mainly affects the tissues with high rates of energy consumption, such as the proximal tubules of the kidneys [37]. Previous studies have shown that mitochondrial autophagy is activated in renal tubular cells [38,39] and that mitochondrial pathology contributes to the development of AKI and can be an early marker of AKI [40,41].

In this study, GSEA of RNA-seq data showed that KDM4C was involved in several pathways, including mitochondrial electron transport chain and oxidative phosphorylation. This suggests that KDM4C may play a role in regulating mitochondrial function. The regulation of mitochondrial gene expression has yet to be well characterized. Transcription factors can regulate the expression of mitochondrial genes indirectly and directly [42]. Although the related research is still limited, the regulatory roles of nuclear transcription factors seem to be the core processes underlying mitochondrial function [43].

Recent studies have suggested that KDM4C may play critical roles in mitochondrial function. The inhibition of KDM4C signaling has been shown to suppress prostate cancer metastasis by interfering with mitochondrial glycolytic metabolism [44]. KDM4A has also been demonstrated to regulate the PDK-dependent metabolic switch between mitochondrial oxidation and glycolysis in tumor cells [45]. Based on these findings, the regulatory roles of KDM4C on the activity of transcription factors may also be crucial for the regulation of mitochondrial dynamics and function. Our findings provide a more detailed molecular understanding of the roles of KDM4C in mitochondrial function regulation.

## 4. Materials and Methods

### 4.1. Zebrafish Study

#### Zebrafish Embryo Staging and Morpholino Injection

Transgenic zebrafish line Tg(wt1b:EGFP) were maintained at 28 °C with a photoperiod of 14 h light and 10 h dark, in an aquarium supplied with fresh water and aeration [46,47]. Embryos were produced using standard procedures and were staged according to standard criteria (hours post-fertilization, hpf) or by days post-fertilization (dpf) [48]. Antisense morpholino oligonucleotides (MOs), *kdm4c*-MO (targeting the 5′-untranslated region and the translation initiation site), were designed and obtained using Gene Tools, Philomath, OR. The MOs were dissolved in 1× Danieau solution containing 0.5% Phenol red, and 2.3 nl of MO solution at the indicated concentration was injected into 1-cell to 2-cell stages of zebrafish embryos.

To examine the phenotypic defects caused by injecting kdm4c-MO, especially defects of the glomerulus and pronephric tube, we used transgenic zebrafish line Tg(wt1b:EGFP) as the test animals18. Ninety-six zebra embryos in the treatment group received a single injection of 0.2 nM MO, and 96 were untreated. Once the embryos were collected, embryo survival, live imaging, and timely observation of renal structures were performed.

See Supplementary Figure S1A for Further Details of the Study Design.

### 4.2. Human Cell Study

#### 4.2.1. Cell Culture and siRNA Plasmid Transfection

HEK293 cells were cultured under conditions suggested by ATCC. H2O2 treatment (at a concentration of 100 mM) was used to induce oxidative stress in vitro. KDM4C knockdown was carried out using KDM4C human siRNA oligo duplex (Locus ID 23081) (Origene, Rockville, MD, USA). JIB04 (Merck & Co., Inc., Kenilworth, NJ, USA) was used to inhibit KDM4C activity. For siRNA plasmid transfection, HEK293 cells were seeded in a 6-well plate (1 × 10^5^ cells/well) and cultured for 24 h at 37 °C. The cells were then co-incubated with 10 µL Lipofectamine^®^ 2000 (Thermo Fisher Scientific, Inc., Waltham, MA, USA) and 4 µg of plasmids at 37 °C for 6 h. Subsequently, the cells were cultured with fresh medium for an additional 24 h. For the autophagy study, serum starvation was performed with serum-free culture.

See Supplementary Figure S1B,C for Further Details of the Study Design.

#### 4.2.2. Flow Cytometry Analysis

Cultured cells were harvested, washed with cold phosphate-buffered saline (PBS), and fixed in 70% ethanol at 4 °C overnight. RNA was removed using Rnase A at 37 °C for 30 min. The cells were then stained with propidium iodide (40 µg/mL) for 30 min at room temperature and analyzed on a FACSAriaIIu_2 flow cytometer (BD Biosciences, Franklin Lakes, NJ, USA).

#### 4.2.3. Western Blotting Analysis

Total protein was extracted using a commercial kit, according to the manufacturer’s instructions (Protein Extraction Kit; Millipore, Burlington, MA, USA). Cell extracts (30 µg protein per lane) were mixed with a sample loading buffer and separated on 12% sodium dodecyl sulfate-polyacrylamide gel. Proteins were electrotransferred onto polyvinylidene fluoride membranes (0.2 µm; Immun-Blot; Bio-Rad, Berkeley, CA, USA). The intensity of each band was quantified using NIH Image software, and the densitometric intensity corresponding to each band was normalized against β-actin expression. The antibodies for Western blotting were KDM4C, LC3B, H3K9M3, BCL1, BCL2, HIF-1α, and actin (Cell Signaling Technology, Inc., Danvers, MA, USA).

#### 4.2.4. Immunofluorescence Staining

Cultured cells were fixed with ice-cold acetone and incubated overnight at 4 °C with primary antibodies (anti-LC3B, Millipore, Billerica, MA, USA). The cells were then incubated with appropriate secondary immunofluorescent antibodies for 1 h at room temperature. The cell nuclei were stained with Hoechst (Molecular Probes, Eugene, OR, USA).

### 4.3. Mice Study

#### 4.3.1. Ischemic Reperfusion Kidney Injury (IRI-AKI) of the Study Animals

Wild-type and *Kdm4c* knockout mice (C57BL/6Ntac-*Kdm4c*^tm1a(KOMP)Wtsi^) were anesthetized with an inhaled anesthesia mixture of isoflurane 2% and oxygen 1 L/min and placed on a temperature-regulated table (39 °C) to maintain body temperature. Renal ischemia was induced by clamping both renal pedicles for 45 min [49]. The sham-operated group underwent the same surgical procedure, except that clamping was not applied.

The study mice were sacrificed at the time points after ischemic reperfusion surgery illustrated (day 3, day 7, day 14, and day 28). The right kidney was harvested on ice and dissected into cortex, outer medulla, and inner medulla regions. Blood samples were collected before ischemic reperfusion surgery. After the operation, blood samples and kidneys were collected at different time points (day 3, day 7, day 14, and day 28) and were processed for different studies (*n* = 8 in each group).

All animal experiments were approved by the Institutional Animal Care and Use Committee of Chang Gung Memorial Hospital (IACUC number: 2008101703, 2012010903, 2021122312). Our animal center is an AAALAC certified center, and this study was performed in accordance with all the relevant guidelines and regulations.

See Supplementary Figure S1D,E for Further Details of the Study Design.

#### 4.3.2. Histopathological Staining

Sections of paraffin-embedded specimens were stained with Masson trichrome, to assess the degree of renal fibrosis. Four random and non-overlapping sections from each kidney specimen were examined under low- and high-power fields.

#### 4.3.3. Cytokine Array Analysis

Kidney protein lysates (100 μg) of the study animal were used for the cytokine analysis with a cytokine array (The Proteome Profiler Mouse XL Cytokine Array Kit, R&D Systems, Minneapolis, MN, USA). Excised tissue was homogenized in PBS with protease inhibitors. After homogenization, Triton X-100 was added to a final concentration of 1%. The samples were frozen at ≤−70 °C, thawed, and centrifuged at 10,000× *g* for 5 min to remove cellular debris. The array procedure and signal intensity calculation were then performed, according to the manufacturer’s instructions.

#### 4.3.4. Quantification of RNA Sequencing

RNA purity and quantification were checked using a SimpliNano™—Biochrom spectrophotometer (Biochrom, Holliston, MA, USA). RNA degradation and integrity were monitored using a Qsep 100 DNA/RNA Analyzer (BiOptic Inc., New Taipei City, Taiwan). A total of 1 μg total RNA per sample was used as input material for the RNA sample preparations. Sequencing libraries were generated using a KAPA mRNA HyperPrep Kit (KAPA Biosystems, Roche, Basel, Switzerland), following the manufacturer’s recommendations, and index codes were added to attribute sequences to each sample.

The library carrying appropriate adapter sequences at both ends was amplified using KAPA HiFi HotStart ReadyMix (KAPA Biosystems, Roche, Basel, Switzerland), along with library amplification primers. PCR products were purified using a KAPA Pure Beads system, and the library quality was assessed using the Qsep 100 DNA/RNA Analyzer (BiOptic Inc., New Taipei City, Taiwan). Library quality was assessed using Qubit@ 2.0 Fluorometer (Thermo Scientific) and Agilent Bioanalyzer 2100 systems. The library was sequenced on an Illumina NovaSeq6000 platform and 150-bp paired-end reads were generated.

The original data obtained from high-throughput sequencing (Illumina NovaSeq 6000 platform) were transformed into raw sequenced reads using the CASAVA base calling and stored in FASTQ format. FastQC and MultiQC were used to check the FASTQ files for quality. The obtained raw paired-end reads were filtered using Trimmomatic to discard low-quality reads, trim adaptor sequences, and eliminate poor-quality bases with the following parameters: LEADING:3, TRAILING:3, SLIDINGWINDOW:4:15, and MINLEN:30 [50,51]. The obtained high-quality data (clean reads) were used for subsequent analysis.

For gene expression, “trimmed mean of m-values” normalization was performed using DEGseq without biological duplicates, and “relative log expression” normalization was performed using DESeq2 with biological duplicates. Differentially expressed gene (DEG) analysis of two conditions was performed in R using DEGseq (without biological replicates) and DESeq2 [52,53,54,55]. The resulting *p*-values were adjusted using Benjamini and Hochberg’s approach, to control the false discovery rate (FDR). Gene ontology (GO) and Kyoto Encyclopedia of Genes and Genomes (KEGG) pathway enrichment analysis of DEGs were conducted using clusterProfiler [56,57]. The DOSE package was used to map disease ontology (DO) terms to MeSH, ICD, NCI’s thesaurus, SNOMED, and OMIM.

Gene set enrichment analysis (GSEA) was performed with 1000 permutations, to identify enriched biological functions and activated pathways from the molecular signatures database (MsigDB) [58,59]. MsigDB is a collection of annotated gene sets for use with GSEA software, including hallmark gene sets, positional gene sets, curated gene sets, motif gene sets, computational gene sets, GO gene sets, oncogenic gene sets, and immunologic gene sets [60]. Weighted gene co-expression network analysis (WGCNA) was used to construct the co-expression network based on the correlation coefficient of expression pattern using the WGCNA package in R [61].

### 4.4. Statistical Analyses

The mean value and standard deviation of data from different animal groups were compared using the Mann–Whitney U test. *p*-values of <0.05 were considered statistically significant.

## 5. Conclusions

In summary, KDM4C may play a vital role in kidney development and AKI. KDM4C is essential for cell survival under stress conditions, by regulating autophagy function. In addition, KDM4C may play a transcription regulation role for mitochondrial function in the kidneys.

## Data Availability

The data presented in this study are available on request from the corresponding author. The data are not publicly available, due to ethical and privacy restrictions.

## Supplementary Materials

The following supporting information can be downloaded at: https://www.mdpi.com/article/10.3390/ijms23169318/s1.
