# Supplementary material for: Essential Roles of the Histone Demethylase KDM4C in Renal Development and Acute Kidney Injury
Source: Int J Mol Sci. 2022 Aug 18;23(16):9318. doi: 10.3390/ijms23169318 (PMC9409075; doi:10.3390/ijms23169318)

Supplementary Figure S1. Study design. (A) Zebrafish study. (B-C) Human cell study. (D-E) Mice study.

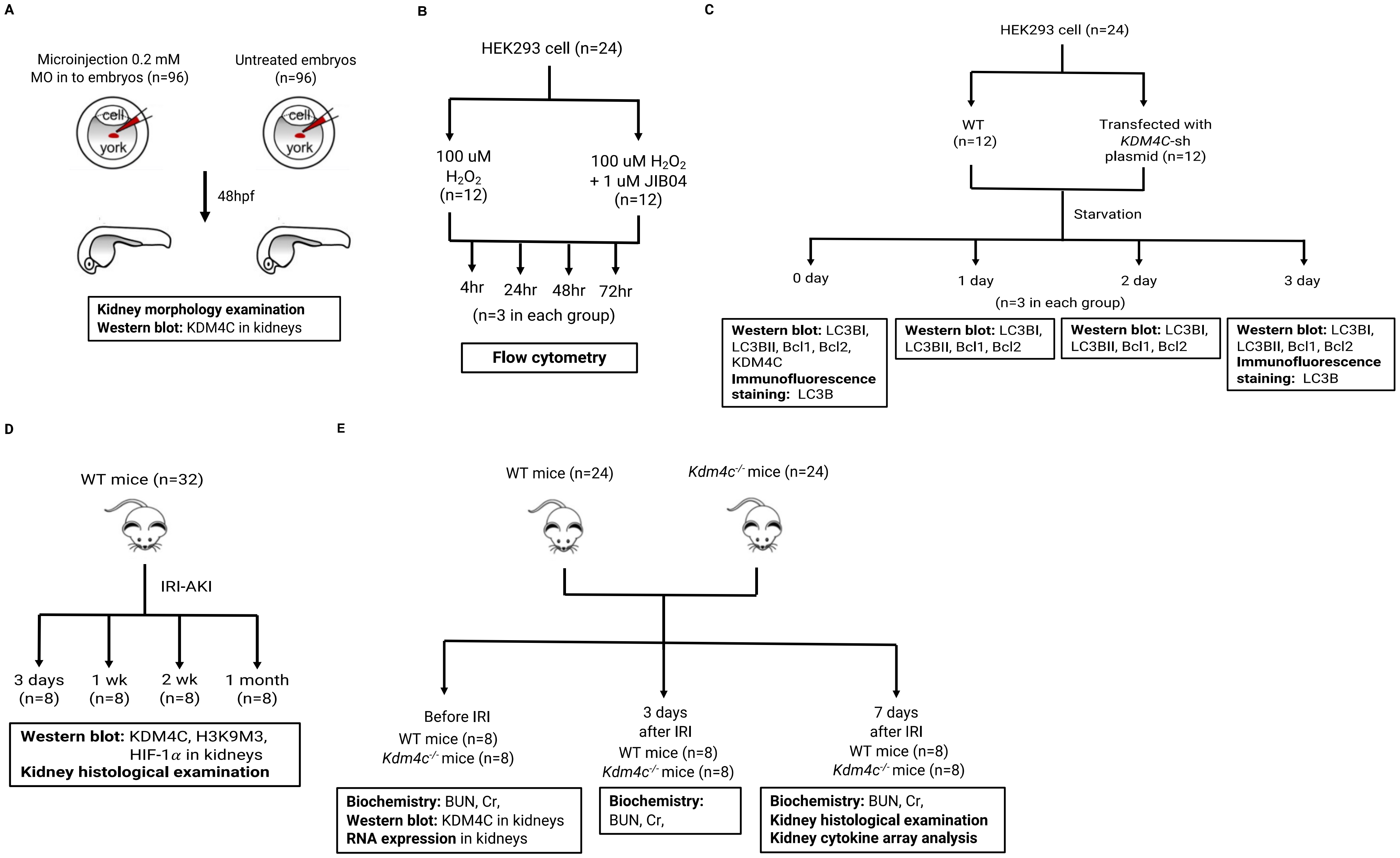

Supplementary Figure S2. Comparison of kidney inflammatory cytokines between wild-type and *Kdm4c*$^{-/-}$ mice. Cytokine array analysis of kidney protein lysates from study animals 1 week after ischemia-reperfusion injury (n=8 in each group).

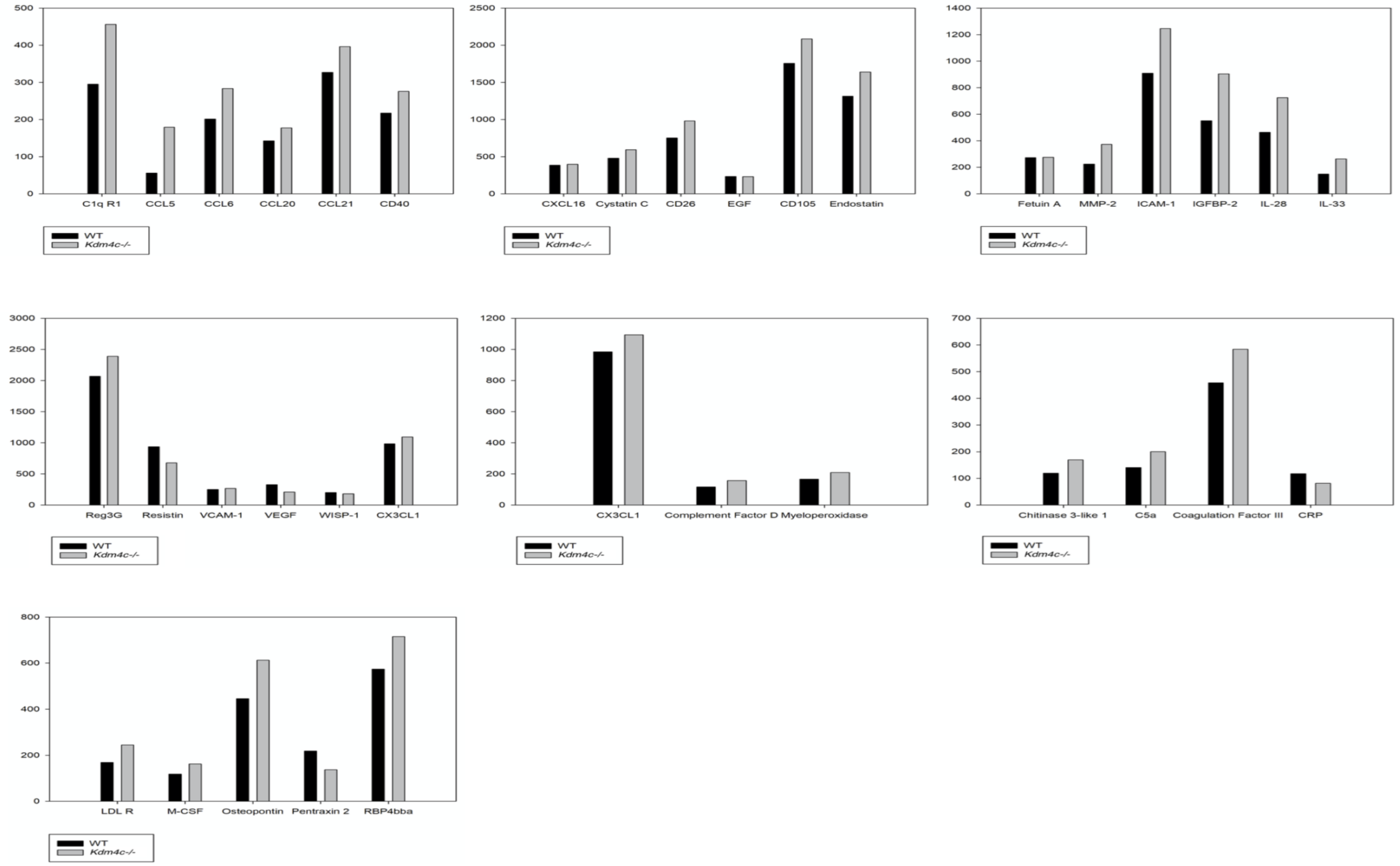

Supplement: Supplementary file 1 [file ijms-23-09318-s001.zip › ijms-1843780-supplementary.pdf]
